# Dr. Hayward’s Correction

**Published:** 1842-10-01

**Authors:** 


### THE MEDICAL EXAMINER.

### PHILADELPHIA, OCTOBER 1, 1842.

Dr. Hayward, of Boston, has written to us to point out an inaccuracy committed by Dr. Clymer, in his paper on "the Mortality of Amputations," published in the thirty-sixth number of the Examiner. Injustice was done to Dr. Hayward and to the Massachusetts General Hospital, by the following statement: "Of 124 amputations reported by Dr. Hayward, of the Massachusetts General Hospital, there were 37 deaths, a fraction more than one in four." Dr. Hayward calls attention to three errors in this statement. He says: "I reported the result of only 70 amputations, that being the whole number that had been performed at the Massachusetts Hospital, up to the time of my report; of the patients in whom three operations had been performed, fifteen only died, being a little more than one in five."

